# Brain effects of nicotine and derived compounds

**DOI:** 10.3389/fphar.2013.00060

**Published:** 2013-05-14

**Authors:** Valentina Echeverria Moran

**Affiliations:** Research and Development, Bay Pines VA Medical CenterBay Pines, FL, USA

**Figure 1:**
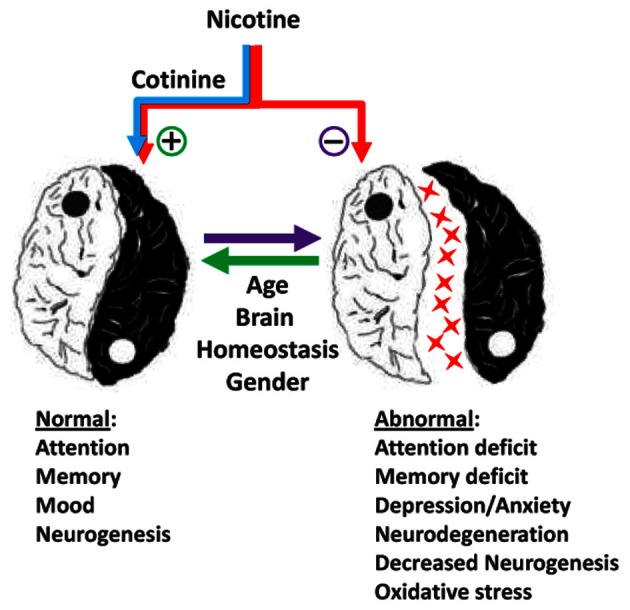
**The Ying and Yang of the effects Nicotine and Cotinine in the adult brain**.

Recent evidence in the field of tobacco research has provided new insights about the mechanisms underlying the effects of nicotine and its derivatives (NAD) in human and animal behavior and pathology. This topic covers several areas of clinical and basic research focused on the effect of NAD on brain function and behavior during development and adulthood. As expressed by Spijker and colleagues (Counotte et al., 2012), there is a Ying and a Yang in the effect of nicotine and its metabolites on the brain (Figure 1).

The influence of NAD on addictive behavior and the mechanisms involved are of great interest for public health. One of our articles explores the molecular mechanisms associated with the differences in addiction to tobacco between genders. They investigated the effect of nicotine, cotinine, and anabaseine on the estrogen biosynthesis in the brain. They show experimental evidence that these compounds affect estrogen synthesis in the brain via the modulation of the last enzyme of the estrogen biosynthesis named aromatase. Based on this evidence they discussed the hypothesis that brain aromatase mediates the effect of nicotine in the brain resulting in sex differences in smoking behavior. Prenatal NAD exposure may affect addictive behavior later in life, several studies have shown that maternal smoking and prenatal nicotine affect brain development and motivated behavior. In here, Harrod et al. show evidence that prenatal nicotine exposure have reinforcing influence over the addictive properties of methamphetamine, affecting vulnerability to addictive behavior in the adulthood (Harrod et al., 2012). Similarly Chen et al. discuss the influence of prenatal nicotine from tobacco smoking on feeding behavior in adulthood as well as the potential of nicotine as a weight loss treatment (Chen et al., 2012). Furthermore, other addictive behaviors such as alcohol dependence may heavily influence the effect of NAD on cognitive abilities. For example, Durazzo et al. found increased neurocognitive deficits in alcohol dependent individuals that also were smokers when compared to non-smokers (Durazzo et al., 2012).

On the other hand, the effect of nicotine on neuroplasticity is controversial, and its improvement and deterioration by NAD have been described. In fact, the investigation by several research groups about the effect of nicotine on attention (Kadir et al., 2006) and learning and memory have given heterogeneous results (Smith et al., 2006). Grundey et al. (2012) show new evidence of a negative effect of nicotine spray on facilitatory plasticity and a diminished reduction in excitability after transcranial direct current stimulation. These results differ from the effects observed after chronic nicotine administration. They attributed these differences to the adaptive nicotinic receptor changes induced by continue nicotine exposure. These heterogeneous effects can be the result of a differential effect of NADs according to the brain state. Counotte et al. (2012) discuss the diverse responses to nicotine on attention, depending on several factors including the extent of exposure (acute vs. chronic), smoking behavior, the developmental stage at which the brain is exposed to nicotine and the presence of psychiatric conditions such as, schizophrenia and Alzheimer's Disease (AD). Psychiatric conditions and/or chronic nicotine exposure may also alter the expression or responsiveness of the cholinergic receptors in the brain and consequently the effect of nicotine on higher order cognitive functions.

Some epidemiological studies have shown data suggesting an inverse relationship between tobacco consumption and the development of AD (Lee, 1994). It has also been found post-mortem that the levels of b-amyloid peptides (Aβ) (considered the neurotoxic agents in AD brains) were significantly decreased in the brains of smoking AD patients compared to non-smokers with the disease. The putative beneficial effect of tobacco has been mainly attributed to nicotine, which has been reported to improve cognitive abilities and reduce plaques in a mouse model of AD (Nordberg et al., 2002). However, nicotine has not demonstrated in clinical studies to be a useful treatment for AD (Lopez-Arrieta et al., 2001). Since nicotinic acetylcholine receptors (nAChRs) play an important role in attention and learning and memory, the positive effects of nicotine on memory have been mostly credited to the activation of these receptors (Sabbagh et al., 2002). A change in the function of nAChRs will influence the release and activity of other neurotransmitters whose release is controlled by these receptors including glutamate, dopamine, serotonin, glycine, and g aminobutyric acid (GABA) (Livingstone and Wonnacott, 2009).

Knott et al. show evidence implicating the N-methyl-d-aspartate receptor (NMDAR) in the beneficial effect of nicotine over auditory, sensory, memory and attention in a human ketamine model of schizophrenia (Knott et al., 2012). In addition, it is discussed the beneficial effect of cotinine, the main metabolite of nicotine, improving memory and attention in several psychiatric conditions including AD (Echeverria et al., 2011), post-traumatic stress disorder (Zeitlin et al., 2012), and schizophrenia (Buccafusco and Terry, 2009). This evidence permits to hypothesize that many of the beneficial effects of nicotine may be at least in part the result of cotinine's actions in the brain (Echeverria and Zeitlin, 2012). The involvement of nicotine action in AD is a complex scientific question, and still needs to be defined whether cognitive impairment in AD is mainly induced by a decrease in the number of nAChRs and/or their function induced by neurotoxic forms of Aβ. In this topic, Zappettini et al. show evidence suggesting that Aβ1-40 inhibits the release of glycine in the hippocampus throughout a mechanism involving the nAChRs but not the muscarinic receptors (Zappettini et al., 2012).

All together this topic gives an actualized view of the NAD effects in aspects of addictive behavior, attention, neuroplasticity, and learning and memory under physiological and pathological conditions.
